# Patterns and Outcomes of dog bite injuries presenting to emergency department in a tertiary care hospital at Karachi

**DOI:** 10.12669/pjms.37.3.3464

**Published:** 2021

**Authors:** Muhammad Ikram Ali, Seemin Jamali, Tehreem Ashraf, Nasibullah Ahmed

**Affiliations:** 1 Dr. M. Ikram Ali, MBBS, DMJ. Assistant Professor, Department of Forensic Medicine, Ziauddin University, Karachi, Pakistan; 2 Dr. Seemin Jamali, Executive Director, Jinnah Postgraduate Medical Centre, Karachi, Pakistan; 3 Dr. Tehreem Ashraf, Ex. House Officer, Ziauddin University, Karachi, Pakistan; 4 Dr. Nasibullah Ahmed Lecturer, Department of Forensic Medicine, Ziauddin University, Karachi, Pakistan

**Keywords:** Dogs, Injuries and Wounds, bites and stings, Animals, Rabies, Post-Exposure Prophylaxis, Zoonoses

## Abstract

**Objectives:**

To assess patterns & outcomes of dog bite injuries coming to a public sector tertiary care hospital in Karachi, Pakistan.

**Methods:**

This was a one-year descriptive cross sectional study from 1^st^ June 2018- 31^st^ May 2019 using consecutive sampling technique. Data of 7512 patients was collected from animal-bite clinic of a tertiary care hospital. Inclusion criteria was animal bite cases that were reported during the dates 1^st^ June 2018 to 31^st^ May 2019, Incomplete records were excluded. Data comprising of time of bite, the location of the victim at the time of bite within the city, animal responsible for the bite, gender and age of victim, date of presentation, site and category of bite (as per WHO criteria) was recorded by the primary investigator. The study was conducted at Jinnah Post-Graduate Medical Centre.

**Results:**

Among 7512 participants 85.8% were males, 32.2% victims reported time of bite between morning and noon, 78.8% of bites involved lower limbs. 51.6% of the bites belonged to category 2. Stray dog bites were observed in 90.3% of cases. Outcome showed 54.9% completed their vaccination, while 44.3% did not show for complete follow up, 3.99% bites were grievous & 0.03% reported with developed rabies.

**Conclusion:**

Research reveals Males belonging to adult age group were most vulnerable, most bites were inflicted in early hours, most common animal inflicting the bites were stray dogs. Many victims did not complete their vaccination from the same centre. Peak of the summer was associated with a decline in number of incidents.

## INTRODUCTION

The association of dogs and humans is incredibly old spreading over 12000 years. Serious health issues related to morbidity are associated with dog bites. The frequency of dog bites occurring in the population of United States of America is somewhere around 500,000 to 4.5 million annually.^1^ Attacks by pet dogs commonly involve head and neck regions, while stray dog attacks involve hands and legs. This variation may be attributed to the difference of attitudes and behaviours towards a pet and a stray animal.^2^

Dog bites, a burden on the health care resources can result in various outcomes. They are considered dangerous because of deadly zoonotic infection rabies, which is the most dangerous incurable outcome.^3^ Rabies is an economic burden for developed as well as developing countries because of the expenses of post exposure treatment, the human suffering and number of fatalities.^4^ The deaths associated with rabies infection attributed to domesticated dog bites by rabid animals are estimated to be around 5900 per year.^5^ In urban regions dog bites play a substantial role in spread of rabies to humans. Recently many cases have been reported from the urban population of Pakistan like Karachi, Peshawar, and Hyderabad.^6^ Pakistan’s government hospitals receive 50 to 70 new cases of dog bite daily.

Rabies is preventable by timely use of post exposure prophylaxis. Awareness regarding rabies prevention is insufficient in most of the developing nations including Pakistan.^7^ Animal and human rabies is prevalent in both urban and rural areas of Pakistan^8^ but unfortunately this issue has never been explored to identify the factors influencing this problem and to design healthcare measures accordingly to address the issue. This study was conducted to study patterns and outcomes in cases of dog bites. This study is will not only identify factors that influence such incidents but will also reflect upon designing the healthcare measures to address the issue.

## METHODS

This is a descriptive cross-sectional study carried out on data collected from a dog bite clinic in a public sector tertiary care hospital using consecutive sampling technique from 1^st^ June 2018 to 31^st^ May 2019 in Karachi. Sample size was n= 7512 cases reported. Data was collected by the principal investigator and comprised of age, sex, location, time of bite, category of bite along with anatomical regions on which bite was sustained by the victim and the animal responsible for the bite, any record with incomplete data was excluded from the study. Ethical exemption was taken from the Ethical Review Committee of Ziauddin University on 5^th^ May 2020 (Reference code: 1940220QHFOR) and the permission to acquire data from the ERC of Jinnah Post-Graduate Medical Centre was taken on 16^th^ April 2019 (Reference No. F 2-81/2019-GENL/19273/JPMC). The study was conducted at Jinnah Post-Graduate Medical Centre.

The SPSS analysis was done on the SPSS version 22. Numerical data was expressed as mean and standard deviation. For categorical data associations were recorded using chi square. P value less than 0.05 was taken as significant. Details such as categorization of bite, age of victims, time and month of bite, site of bite, development of rabies, completion of vaccination from the same centre were taken, analysed, and compared.

According to age the victims were divided into four categories, Children (1 to 10 years of age), Adolescents (11 to 19 years of age), adults (20 to 60 years of age) and old aged being 61 years or more. Time of the bite was also divided into four categories in the following manner, morning to noon (between 06:00 AM to 12:00PM), noon to evening (in between 12:00 PM to 06:00PM), noon to night (in between 06:00 PM to 12:00AM) and night to morning (in between 12:00 AM to 06:00AM).Time of bite was known to the exact hour in n=3586 (47.7%) of cases that reported within 24 hours of bite in the rest of the cases n=3926(52.3%) only date of the bite was recorded as they presented to the clinic after 24 hours.

The categorization of the wound was done according to WHO defined criteria with bites being categorized in three categories, Category-I: touching or feeding animals, animal licks on intact skin (no exposure), Category-II: nibbling of uncovered skin, minor scratches with skin breeched, or abrasions without bleeding (exposure) Category-III: single or multiple transdermal bites or scratches, contamination of mucous membrane or broken skin with saliva from animal licks, (severe exposure).

## RESULTS

Among n=7512 participants n=6444 (85.8%) were males and n= 1068(14.2%) were females. Among male victims majority of the animal bites involved the lower limbs n=5121(79.5%) followed by bites inflicted on upper limbs n=925(14.4%). In females lower limbs were injured in n=790(74%) of the cases while upper limb injuries were seen in n=169(15.8%) of the cases. (p value 0.000). Among male victims n=5912(91.8%) bites were inflicted by stray dogs, followed by pet dogs n=211(3.3%). Among female victims n=875(82%) bites were inflicted by stray dogs followed by pet dogs n=64(6%) (p value 0.000).

About 6787(90.3%) bites were inflicted by stray dog attacks while pet dog bites were n=275(3.7%) rest of the bites being attributed to attacks by other animals. In all age categories it was noticed that males were far more affected by dog bites as compared to females as shown in Graph-1, that also compares time of bite with gender.

**Fig-1:**
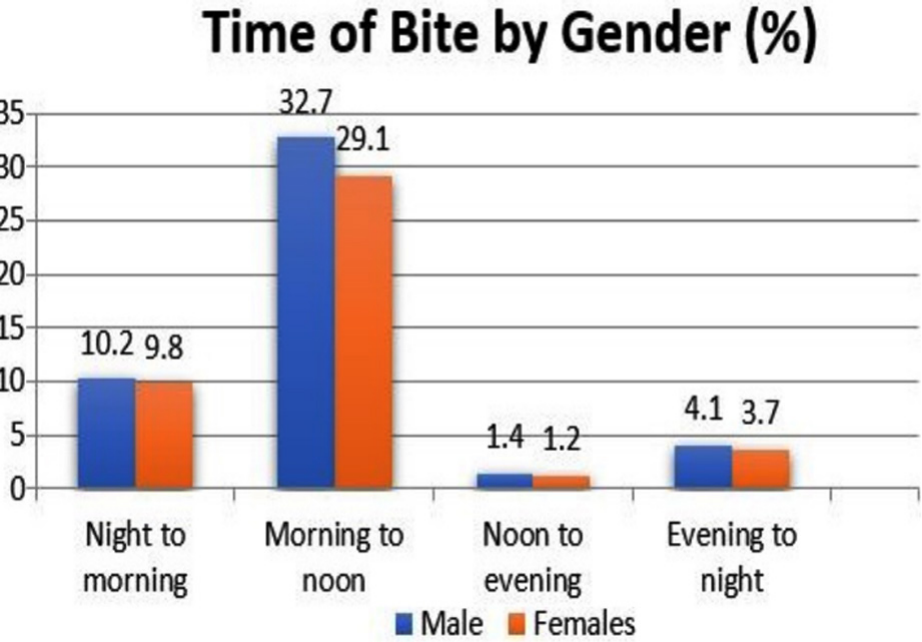
Comparison of time of Bite & Gender.

Among different age categories it was noted that most victims, n=4456(59%) belonged to the adult age group while old, aged individuals were least victimized n=435(0.05%) by the animals. In all age categories lower limbs were maximally affected. N=828 (66.3%) of the children sustained bite injuries on the lower limbs, among adolescents n=1067(77.7%) sustained bite on the legs, among adults n=3657(82.1%) were bitten on lower limbs and among the old age n=359(82.5%) sustained injuries on lower limbs. (p value 0.000).

When different age groups were analysed according the animal that was responsible for the bite it was observed that children n=1156(92.6%) sustained bite injuries because of a stray dog attack followed by n=40(3.2%) because of pet dog attack. Among the adolescents n=1260(91.8%) were attacked by stray dogs while n=52(3.8%) were bitten by a pet dog. Among adult age group most bite injuries n=3987(89.3%) were attributed to a stray dog attack followed by pet dogs n=170(3.8%). Among old, aged population most of the bites were cause by stray dogs, n=393(90.3%) followed by pet dogs n=13(3%). Among children n=26(2.1%) had head bites, n=43(3.4%) had neck & face bite (p value 0.000).

Bite category stratification revealed, out of n=7408 bites n=63 (0.8%) were Category-I bites while n=3812 (51.4%) were Category-II and n=3533(47.6%) were Category-III bites, a detailed comparison of categories of bites according to different age groups is shown in Table-I.

**Table-I:** Category of bite (as classified by who criteria) compared to age groups.

Age Category	Category-I Intact skin licked		Category-II Scratch & breech of skin		Category-III Multiple, transdermal bites		P Value

	N	%	N	%	N	%	
Children (n=1248)	12	1	521	41.7	715	57.3	0.0000
Adolescents (n=1373)	6	0.4	696	50.6	671	48.8	
Adults (n=4456)	41	0.9	2386	53.6	2029	45.5	
Old Age (n=436)	4	0.9	209	48	222	51	

The exact time of bite was known in n=3586(47.7%) while it was not recorded in n=3926(52.3%) of the cases as they reported after 24 hours. Maximum patients reported the time of bite between morning to noon n=2418(32.2%) while minimum cases of bite were seen between noon to evening n=101 (1.3%). Time slot between evening to night displayed n=302 (4%) while duration between night to morning showed n=765(10.2%) of cases. A detailed comparison of time of bite with different age groups is displayed in Table-II.

**Table-II:** Time of bite by age group of patients.

Age Category	Night to Morning (between 12:00 AM to 06:00AM).		Morning to Noon (between 06:00 AM to 12:00PM)		Noon to evening (between 12:00 PM to 06:00PM)		Evening to Midnight (between 06:00 PM to 12:00AM)		Time of bite not known (Reported after 24 hours)		P value

	N	%	N	%	N	%	N	%	N	%	
Children (n=1248)	115	9.2	415	33.3	24	1.9	39	3.1	655	52.5	
Adolescents (n=1373)	118	8.6	430	31.3	22	1.6	54	3.9	749	54.6	
Adults (n=4456)	487	10.9	1420	31.9	50	1.1	196	4.4	2303	51.7	0.089
Old Age (n=435)	45	10.3	154	35.4	5	1.1	13	3	218	50.1	

Majority of cases n=939 (12.5%) were seen in April whereas least cases were reported in May n=389(5.2%). Other months’ showing a higher rate of dog bites are March, n=895(11.9%) and February, n=821(10.9%) while months showing comparatively less frequency are July n=524(7%) and June n=562(7.5%). Maximum bites n=570(7.5%) were reported from Malir, followed by, n=280 (3.7%) from Landhi, n=249(3.3%) from Korangi and n= 179(2.3%) bites reported from Baldia Town.

Around n=4126(54.9%) victims completed their vaccination from the same centre while n=3329(44.3%) did not complete vaccination. As far as the outcomes and status of completion (Vaccine) is concerned the detailed analysis is displayed in Table-III.

**Table-III:** Outcomes of patients presented with dog bite.

Total Number of Patients		Patients that completed vaccination from the same Centre		Patients that started but did not complete the vaccination		Patients with vaccination status not known		Patients presenting with Rabies		Patients with grievous & difficult to treat injuries involving head, face & neck, chest & abdomen, & Multiple bites.	

N	%	N	%	N	%	N	%	N	%	N	%
7512	100	4126	54.9	3329	44.3	57	1.7	3	0.03	300	3.9

## DISCUSSION

Amongst all animals, bites inflicted by dogs form the greatest burden on the healthcare resources and domesticated dogs are the topmost reason behind rabies spread.^9^,^10^ In underdeveloped countries this burden is further increased because of the inaccessibility to the vaccine and its cost.^11^ This study is compared to previous studies done in both developed and underdeveloped countries to identify different aspects of the problem and to explore the factors having potential to modify the magnitude of the issue.

In developed regions of the world it has been noted that females, teenagers and children are more likely to be victim of dog bites as compared to males^12^,^13^ and other age groups, however in our study it was observed that males were mostly bitten 85.7% while the rest of the victims 14.2% were females, probably because females in Pakistan usually stay at home due to the social and cultural reasons. While children comprised around 16% of victims that reported to the hospital. The highest number of cases being attributed to the adult age group being 59%. A previous similar three-year study carried out in the rural setting of Punjab^4^ another province of Pakistan, showed that similar findings were observed as have been noticed during our study like a greater number of victims belonged to the male gender, most cases were reported with lower limb injuries and 80% of the bites being categorized as belonging to the categories II and III. Their study was different as greater number of victims being involved were children while in our study most victims belonged to the adult age group. The variations can be attributed to social and cultural behaviors and can be influenced by the attitude of the urban and rural population towards animals.

Stray dog bites carry a high risk of infection as these are unvaccinated dogs 14.^15^ posing a greater threat in comparison to the pet dogs which have been responsible for most bites in the more developed regions of the world.^7^,^16^ In Pakistan most of the bites are by stray animals rather than the ones that are kept as pets probably because of the number of the stray dogs being greater than those kept as pets.

In a study taking place in China it was noted that most of the bites categorized according to WHO prescribed criteria as Category-II and Category-III bites^10^ while very few less than 4% of the bites reported as Category-I bites. Similar findings were observed in the categorization of bites in our study i.e., only 0.8% of the bites belonged to Category-I, while 51.4% of the bites were categorized as Category-II and 47.6% bites were belonging to Category-III. Most probably because Category-I bites do not involve any breach in skin are never taken as bites by the victims and are ignored.

Peak number of cases reported in the month of April followed by March and February while it was also seen that substantially smaller number of cases reported during the months of June and July when the peak of the warm weather is experienced in Karachi, unlike in other studies conducted in United states and Korea where maximum cases were observed in the warm season especially in the month of May^17^ a finding different to the observations in our study. Greatest number of dog bites in the study in Punjab^4^ took place during the months of May, June and July, while in our study greatest number of cases were reported in the months of April, March, February. An observation in contrast to the previous studies done in Pakistan is that the peak of bite incidents were seen in summers while the peak of summers is observed in Karachi in June & July months in which less numbers of cases are reported. A fact that can further be explored to identify the reasons.

During our study we identified certain regions within the city where the incidence of dog bite was higher. Vaccination is available free of cost only in very few government run or charity based medical facilities, a fact that attributes for such a large number of patients reporting to the hospital where this study was conducted, controlling the population of stray dogs within the city, providing more animal bite clinics in the areas displaying the greater number of cases with enhancing the public awareness regarding this preventable healthcare issue is the way to proceed forward in addressing this problem.

Another important observation related to outcome of patients reporting late, the frequency of such cases being almost 52.3% in our study, along with 54.9% of the reported patients completing their vaccination from the same animal bite clinic while the others either continued their vaccination in some other hospital or discontinued, discontinuation can be associated with the lack of awareness within the population^18^ a problem that raises serious concerns. Another finding to be noted was that three patients developed rabies and died: a condition preventable but untreatable in its course as shown in Table-III.

### Strength and Limitations of the study:

It was conducted in Karachi a city of Pakistan that has been highlighted for its maximum number of cases in comparison any other region in Pakistan.^6^ The data retrieved in this study was from public sector hospital with the greatest number of patients reporting not only from the city but also from the surrounding rural areas. Some of the limitations of the study were related to not including other tertiary care government run hospitals situated with in Karachi providing treatment to dog bite victims.

Before this study was submitted to be published it was cited in some national newspapers the cases of dog bite increased in frequency and as per news it was revealed that the turnover of patients increased to 630 victims per day a very high number in comparison to the one observed in our study with multiple factors such as increased population of stray dogs and administrative deficits.^19^ Similar news was published in another newspaper highlighting same situation but focused on the aspect of sudden surge in rabies cases also revealing the acute shortage of the rabies vaccine and its unavailability throughout the province of Sindh.^20^

## CONCLUSION

Research reveals Males belonging to adult age group were most vulnerable, most bites were inflicted in early hours, most common animal inflicting the bites were stray dogs. Many victims did not complete their vaccination from the same centre. Peak of the summer was associated with a decline in number of incidents.

### Authors’ Contribution:

**IA:** Besides being responsible for the accuracy and integrity of the work, conceived the idea, designed the study, and was involved in finalizing the draft.

**SJ:** Helped in acquisition of data revised the article identifying key parameters to assess. She is also responsible for the accuracy and integrity of the data.

**TA & NA:** Helped in data acquisition and statistical analysis.

**TA:** Was also involved in literature review.
